# Behavior Based Social Dimensions Extraction for Multi-Label Classification

**DOI:** 10.1371/journal.pone.0152857

**Published:** 2016-04-06

**Authors:** Le Li, Junyi Xu, Weidong Xiao, Bin Ge

**Affiliations:** College of Information System and Management, National University of Defense Technology, Changsha, Hunan, P.R. China; Universidad Veracruzana, MEXICO

## Abstract

Classification based on social dimensions is commonly used to handle the multi-label classification task in heterogeneous networks. However, traditional methods, which mostly rely on the community detection algorithms to extract the latent social dimensions, produce unsatisfactory performance when community detection algorithms fail. In this paper, we propose a novel behavior based social dimensions extraction method to improve the classification performance in multi-label heterogeneous networks. In our method, nodes’ behavior features, instead of community memberships, are used to extract social dimensions. By introducing Latent Dirichlet Allocation (LDA) to model the network generation process, nodes’ connection behaviors with different communities can be extracted accurately, which are applied as latent social dimensions for classification. Experiments on various public datasets reveal that the proposed method can obtain satisfactory classification results in comparison to other state-of-the-art methods on smaller social dimensions.

## Introduction

Due to the wide applications in fraud detection [1], gene family prediction [2] and counterterrorism analysis [3], the research of within-network classification has been very active in recent years. Given a partially labeled network, in which labels of some nodes are known, within-network classification aims to predict the labels of rest nodes. As nodes in network are interconnected, relational classification methods can make use of the connectivity information to predict unknown nodes. For example, the labels of neighbor nodes are of high correlation, so unknown nodes can be predicted via a weighted average of the estimated class membership of the node’s neighbors [4, 5]. Topology structure can also provide valuable information for classification, so similarity measures (such as random walk, common neighbors, etc.) are used to predict unknown nodes by estimating the structure similarity with labeled nodes [6–8]. By exploiting network connectivity information, all above methods are shown to have satisfactory performance on single label classification task, which assumes node is only associated with one label.

However, multi-label classification task, where a node can be associated with more than one labels, is becoming a new challenge recently. In single label classification task, edges in the network are treated homogeneously; the implicit assumption is that the edges are engendered from similar processes [9]. While in multi-label network, the interactions between nodes are driven by various reasons. For example, in social networks, a woman who loves pets and a man who loves photograph will focus on the same person who is a pet photographer, and at the same time, the pet photographer may connect with his friend who is a programmer. The information of the edges is often difficult to be obtained, so it is very important to identify the diversity connections in such a situation. Many existing approaches ignore the different types of edges and treat these relations homogeneously, leading to a lower classification performance [10].

In order to handle the network heterogeneity, instead of using explicit edges, researchers try to mine the implicit relationships between nodes in various ways [6, 9, 11]. SocioDim [10] is one of the representative classification frameworks. Given a partially labeled network, SocioDim performs community detection algorithm on the network at first, and uses the community memberships as the latent social dimensions. Then discriminative classifiers (e.g. logistic regression, support vector machine) are applied to predict the unknown nodes.

**Fig 1:**
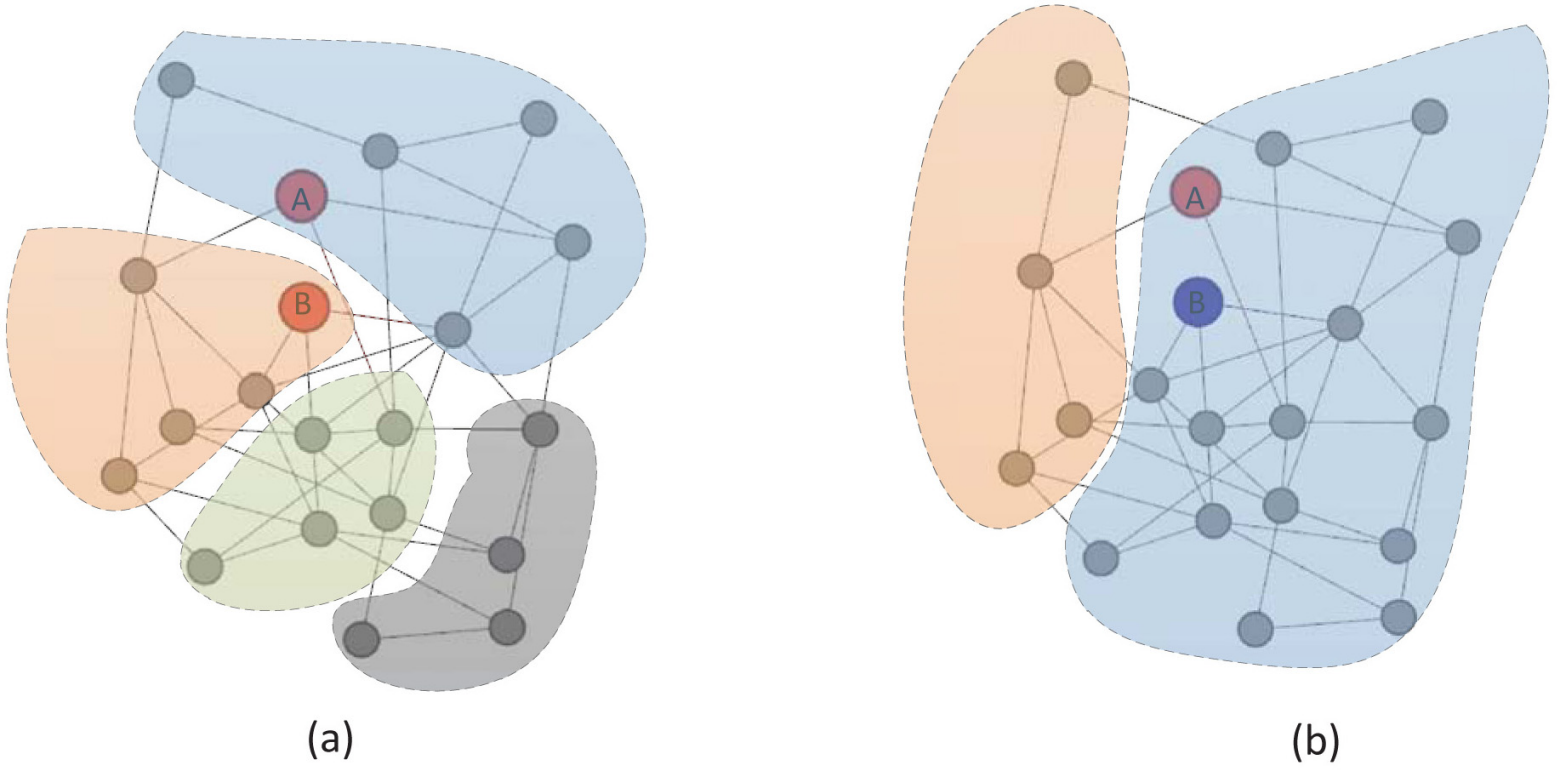
Classification process of SocioDim. The color of node A and node B represents their label. The shadow area represents the community, and different colors represent different communities. For soft clustering, the figure shows the results exceeding a threshold.

However, as we know, the performance of community detection algorithms is affected by many factors, such as network properties and parameters of community detection algorithms [12–14]. When community detection algorithms fail, social dimensions cannot be extracted accurately and result in lower classification performance. Fig 1(a) shows the result of a community detection algorithm, which is not suitable for the current network. As it can be seen, node A and node B have the same label, but are assigned to different communities. In this case, using nodes’ membership in communities cannot extract social dimensions accurately and leading to poor performance. Fig 1(b) shows a community detection result, in which the parameters are set incorrect. In this situation, many small communities will merge into large communities, making the communities contain a large number of nodes with different labels. Community detection based methods tend to identify these nodes with similar social dimensions, so that the classification performance drops.

In addition, in order to handle classification task on large-scale networks, it often requires high-performance computers to make prediction. By mining latent social dimensions, the features of nodes can be reduced to smaller dimensions, making an ordinary computer meet the requirement of classification in large-scale networks. However, existing methods still require relatively larger dimensions to achieve better results [15]. Therefore, we also focus on how to use smaller social dimensions to obtain satisfactory classification performance.

In this paper, we propose a novel social dimension extraction method, BBSD (Behavior based SocioDim), to handle multi-label classification task in the network. Instead of relying on the community detection method to extract social dimensions, our method tries to identify nodes’ interaction behavior with different communities, and then extract the implicit social dimensions by analysis of behavior features. We prove that BBSD has a strong fault-tolerant ability for community detection algorithm, which can effectively improve the classification performance when some community detection algorithms fail. In addition, in the case of limited computing resources, our method can extract refined social dimensions to achieve satisfactory performance in the classification task.

## Related Works

In heterogeneous networks, edges are driven by various reasons, but the related information is often difficult to be obtained. Therefore, only applying observed connections is not enough for handle multi-label classification task. Mining latent relationship is a kind of representative methods, which aim to find the latent relationship between labeled nodes and unknown nodes. Probabilistic relational models [16–18] can construct the dependence between connected nodes. The probability of an unknown node’s label is conditioned not only on the labels of neighbors, but also on all observed data. By building the probability dependence in the network, this kind of methods can avoid the impact of various relations, and handle the classification task in heterogeneous networks effectively. However, computing complexity of these methods becomes a barrier for practical application in large-scale networks [10, 19].

Similarity-based classification techniques have been proposed to handle this problem. By performing random walk on the network, Gallagher et al. [6] can estimate the distance between labeled nodes and unlabeled nodes. Then the distance can be used as weighted of “ghost edges” to predict the unknown nodes. In addition, they use an even-step random walk with restart to handle the network heterogeneity, which can overcome the impact of heterogeneous networks effectively. Zhang et al. [7] apply several similarity-based link prediction methods for network classification task. By comparing the similarity of nodes, they are able to find latent relationship between labeled nodes and unknown nodes, and weights are calculated according to different similarity indices.

Extracting latent group is another kind of methods for handling the network heterogeneity. Given a network, if there is an edge between node *x* and node *y*, it can be considered that they are connected directly. However, in network generation process, the connection behavior between node *x* and node *y* is complex, rather than a direct connection process. For example, in social networks, user *x* wants to follow an expert in *java*. But in most cases, he does not know who will be the expert in the field of *java*. He may search the community discussing *java* at first, and find the experts often express comments in the community. Then he randomly chooses an expert *y* and follows him. Therefore, constructing the relationship between communities and nodes will be valuable for the classification process. Latent group model (LGM)[11] is proposed to utilize the hidden structures responsible for the observed autocorrelation among labels. However, LGM gets the hard cluster membership of each node, which will be easily affected by the noisy data [10]. By introducing more complex relationships dependency, nonparametric relational model (IHRM) is used to model and analyze social networks [20], which can consider relations between members in all groups. However, both methods are based on generative model, and high computational cost for parameter inference makes them not suitable for classification in large-scale networks [19].

SocioDim is another representative classification framework by utilizing latent group. The core idea is to extract the latent social dimensions by community detection methods. Social dimensions can be treated as new features of nodes, and then discriminative classifiers are used to predict unknown nodes. Tang et.al [10] use spectral clustering to extract social dimensions and apply Support Vector Machine (SVM) for classification, which has shown better classification performance. Modularity maximization [19] is a typical community detection method, which can also be applied to extract social dimensions, but the computation complexity is too high to be applied in large-scale networks. EdgeCluster [15] is another novel method which uses edge-centric k-means clustering to obtain sparse social dimensions. It can handle classification task on networks which are too large for spectral decomposition, and obtain comparably performance to modularity maximization method. DeepWalk [21] applies the popular deep learning method to extract social dimensions and get much better results, but high computing resources is usually needed in deep learning method. Wang and Sukthankar [9] use EdgeCluster to extract social dimensions. Then, instead of using SVM as discriminative classifier, they design the multi-label iterative relational neighbor classifier (SCRN) to handle the multi-label classification task, which significantly boosts classification performance on collaboration networks.

## Behavior based Social Dimensions Extraction

In this section, we introduce the intuition of behavior based social dimensions extraction at first, and then we will describe LDA based network generation process to obtain behavior features. Finally, the differences between our method and LDA based community detection methods are explained in detail.

### Intuition of Behavior based Social Dimensions Extraction

SocioDim is an effective framework to handle the multi-label classification task in heterogeneous networks. However, most classification methods based on SocioDim use community detection to extract latent social dimensions [9, 10, 15, 19], making the classification ability rely heavily on the performance of community detection algorithms. To overcome this problem, we aim to utilize other strategies to extract the social dimensions.

Community detection methods focus on extracting the social dimensions based on the principle of maximum modularity, etc. From the perspective of community detection, the network topology is static. However, from the perspective of network evolution, the existing network is generated by the interactions between nodes. In the network generation process, the interaction behaviors and label of nodes are of high correlation [9, 10], i.e. nodes tend to interact with different communities based on their labels (such as interest, gender, etc.). For instance, the persons with “pet” label tend to follow veterinarian and pet photographer. Therefore, an intuition is that we can utilize behavior feature to predict the label of nodes. When community detection methods yield poor performance, nodes with similar labels may be assigned to different communities. In this case, community detection based methods will fail to extract social dimensions. Compared to node’s community membership, using behavior feature to extract social dimensions is less affected by the performance of community detection algorithms. When community detection algorithms fail, using behavior feature is stable to extract the latent social dimensions accurately and obtain more satisfactory results.

As shown in Fig 1(a), node A and node B have same labels, but are assigned to different communities, community detection based methods cannot make accurate prediction. However, it can be found that for node A and B, the community distributions of neighbor nodes are the same. In this case, node A and node B have the similar behavior features. Our method tends to predict the same labels for node A and B, and obtains better performance. For Fig 1(b), node A and node B have different labels, but are assigned to the same community, community detection based methods tend to make the wrong prediction. However, it will be easy to find that node A makes interaction with two communities, while node B only interacts with the largest community. Considering the community distributions of neighbor nodes, node A and node B have different behavior features. Our method tends to predict different labels for node A and node B, and gets satisfactory results.

Community detection based methods use node’s community membership for classification, which may yield poor performance when community detection algorithms fail. However, our method uses node’s behavior feature (community distributions of neighbor nodes) for classification. Compared to using the community distribution of only one node (node itself), our method takes advantage of all neighbor nodes’ community distributions to describe the feature of the node. The probability of incorrect community assignment of all the neighbor nodes is much lower, so using community distributions of neighbor nodes will be less affected by the performance of community detection algorithms. When community detection algorithms perform poorly, our method has strong fault-tolerant ability, which can still extract the social dimensions accurately.

### Mining Behavior Feature based on LDA

The network is generated by interactions between nodes, so we need to find a suitable method to model the generation process. Probabilistic generative models have been successfully used for modeling text and networks, where LDA is one of the most famous models [22]. By modeling the generation process on text, LDA can inference the latent variable: topic, and obtain probability distributions of document-topic and topic-word.

We apply the idea of LDA to model the network generation process, and try to mine latent behavior features between nodes and communities. Then we treat these behavior features as latent social dimensions to handle classification task.

We assume that the generation process for a directed weighted network is as follows:

1. According $\vec{\beta }$ prior distribution, generate *K* mixture components:${\vec{\varphi }}_{k}∼Dir\left(\vec{\beta }\right)$, representing *K* communities.
2. For each node *m* ∈ [1, *M*] in the network,
  - According $\vec{\alpha }$ prior distribution, generate ${\vec{\theta }}_{m}∼Dir\left(\vec{\alpha }\right)$, representing node *m*’s connecting probability with different communities.
  - Node *m* makes *N*_*m*_ connections, the *n*-th connecting behavior is:
    - Choose community $z_{m,n}∼Mult\left({\vec{\theta }}_{m}\right)$,
    - Choose node $c_{m,n}∼Mult\left({\vec{\varphi }}_{z_{m,n}}\right)$ to connect, *w*(*m*, *c*_*m*, *n*_) = *w*(*m*, *c*_*m*, *n*_)+1.

Where *w*(*m*, *c*_*m*,*n*_) is the weight of the edge from node *m* to node *c*_*m*,*n*_. ${\vec{\varphi }}_{k}$ is the probability distribution of nodes belonging to the community *k*, and distributions of all communities are $\underset{}{\underline{\varphi }}={\left\{{\vec{\varphi }}_{k}\right\}}_{k=1}^{K}$ (*K* × *M* matrix). ${\vec{\theta }}_{m}$ is node *m*’s probability distribution of connecting with different communities, and distributions of all nodes are $\underset{}{\underline{\theta }}={\left\{{\vec{\theta }}_{m}\right\}}_{m=1}^{M}$ (*M* × *K* matrix)

Given the hyperparameters $\vec{\alpha }$ and $\vec{\beta }$, the joint distribution of node *m*’s connection behavior is:

$$p({\vec{c}}_{m},{\vec{z}}_{m},{\vec{\theta }}_{m},\underset{}{\underline{\varphi }}|\vec{\alpha },\vec{\beta })=\prod_{n=1}^{N_{m}}p(c_{m,n}|{\vec{\varphi }}_{z_{m,n}})p(z_{m,n}|{\vec{\theta }}_{m})p({\vec{\theta }}_{m}|\vec{\alpha })p(\underset{}{\underline{\varphi }}|\vec{\beta }).$$ (1)

Where ${\vec{z}}_{m}$ is the communities sequence obtained in the process of node *m*’s connection behaviors (*n*-th connected community is *z*_*m*,*n*_), ${\vec{c}}_{m}$ is the nodes sequence (*n*-th connected node is *c*_*m*,*n*_).

Gibbs sampling [23], a special case of Markov-chain Monte Carlo (MCMC) simulation, can be used to make inference approximately. Given a network, the connection behavior of all nodes $\vec{z}$ and $\vec{c}$, are the parameters need to be sampling. As pointed in [24], the joint distribution of $\vec{z}$ and $\vec{c}$ is:

$$\begin{array}{c} p\left(\vec{z},\vec{c}|\vec{\alpha },\vec{\beta }\right)=\prod_{m=1}^{M}\frac{\Delta ({\vec{n}}_{m}+\vec{\alpha })}{\Delta (\vec{\alpha })}\prod_{k=1}^{K}\frac{\Delta ({\vec{n}}_{k}+\vec{\beta })}{\Delta (\vec{\beta })}. \end{array}$$ (2)

Where $n_{m}^{\left(k\right)}$ refers to the number of times that node *m* has been observed to connect with community *k*, ${\vec{n}}_{m}={\left\{n_{m}^{\left(k\right)}\right\}}_{k=1}^{K}$. $n_{k}^{\left(t\right)}$ denotes the number of times that node *t* has been observed in community *k*, ${\vec{n}}_{k}={\left\{n_{k}^{\left(t\right)}\right\}}_{t=1}^{M}$.

Therefore, the update equation for the hidden variable can be derived:

$$\begin{array}{c} p\left(z_{i}=k|{\vec{z}}_{\neg i},\vec{c}\right)\propto \frac{n_{m,\neg i}^{\left(k\right)}+\alpha_{k}}{\sum_{k=1}^{K}n_{m,\neg i}^{\left(k\right)}+\alpha_{k}}\cdot \frac{n_{k,\neg i}^{\left(t\right)}+\beta_{t}}{\sum_{t=1}^{M}n_{k,\neg i}^{\left(t\right)}+\beta_{t}} \end{array}$$ (3)

Where count $n_{\cdot ,\neg i}^{\left(\cdot \right)}$ means the connection behavior *i* is excluded from the current calculation process.

Then parameter set *θ*_*m*,*k*_ (the probability of node *m* connects with community *k*) and *φ*_*k*,*t*_ (the probability of node *t* belongs to community *k*) can be obtained according to following equations.

$$\begin{array}{c} \theta_{m,k}=\frac{n_{m}^{\left(k\right)}+\alpha_{k}}{\sum_{k=1}^{K}n_{m}^{\left(k\right)}+\alpha_{k}} \end{array}$$ (4)

$$\begin{array}{c} \varphi_{k,t}=\frac{n_{k}^{\left(t\right)}+\beta_{t}}{\sum_{t=1}^{M}n_{k}^{\left(t\right)}+\beta_{t}} \end{array}$$ (5)

Eq (4) represents the probabilities that nodes’ connection behavior with different communities, Eq (5) represents the probabilities that nodes’ membership in different communities. As it can be seen, Eq (4) is the behavior feature of nodes, and we use it as social dimensions for handling classification task.

The runtime complexity of standard Gibbs sampling [25] is *O*(*MKD*). *M* is the number of nodes, *K* is the number of communities, and *D* is the average node degree in the network. The space complexity for both is *O*(*N*(*K* + *D*)).

### Difference with LDA-based Community Detection Methods

Through the analysis of document generation process, LDA can extract latent topic distribution in the documents, where a topic is defined as a distribution over a fixed vocabulary of terms. As communities in network are also implicit, many researchers applied LDA to handle community detection task [25, 26]. Both BBSD and LDA-based community detection (LDA-CD) methods use LDA to model the network generation process, but there are obvious differences.

**Fig 2:**
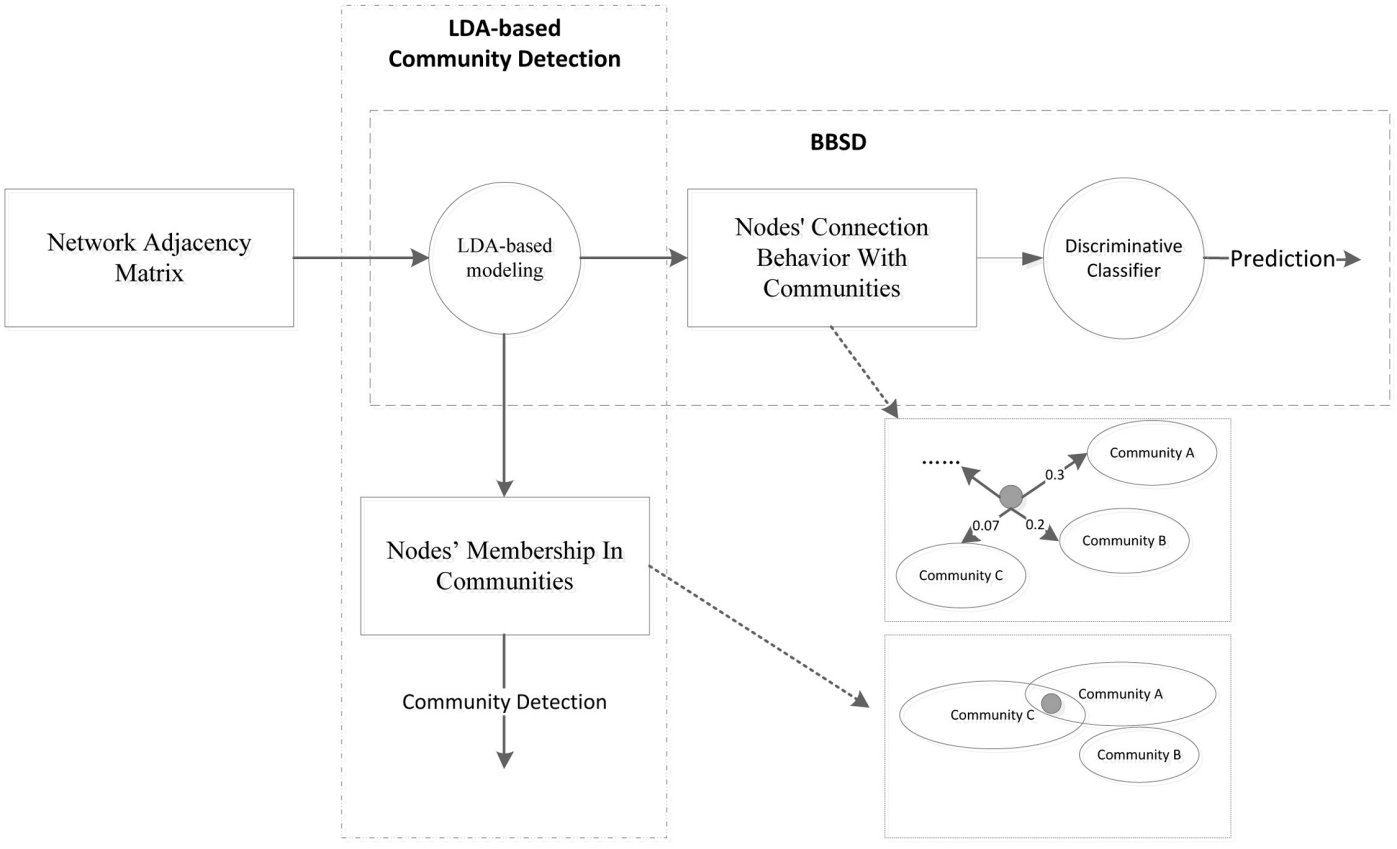
Difference between BBSD and LDA-CD.

Firstly, we aim to extract the behavior feature between nodes and communities, which is shown in Eq (4), while the LDA-CD tries to mine the probability of nodes’ membership in communities, which is shown in Eq (5). Secondly, we use behavior features as latent social dimensions to deal with multi-label classification problem in heterogeneous networks, while LDA-CD focuses on community detection task, rather than classification task. Thirdly, traditional LDA based community detection methods generally require many pre-processing operations to get better results (for example, converting original network to K-times collaborative networks [26]), while our method does not require any pre-processing operations. Finally, in the experiment, we also apply LDA-CD to extract social dimensions, which are used as a baseline method. We demonstrate that when using LDA to model the network, behavior feature based classifier (BBSD) performs better than community detection based classifier (LDA-CD) in default parameters. Fig 2 shows differences between BBSD and LDA-CD.

## Experimental Setup

In this section, we introduce four baseline methods for comparison with BBSD, and then we describe the datasets collected for experiments. Finally, two evaluation measures: Macro-F1 and Micro-F1 are explained in detail.

### Baseline Methods

We compare BBSD with two community-detection based social dimensions extraction methods (LDA-CD [25, 26] and EdgeCluster [15]), and two baseline methods without learning (a Majority Model and a Random Model).

#### 1. LDA-CD

LDA-CD can be used to perform community detection on the network [25, 26]. We introduce LDA-CD into SocioDim framework, and use it to extract latent social dimensions. To get better community detection result, pre-processing operations (e.g. converting original network to K-times collaborative networks) are required to transform the network. However, we directly applied LDA-CD on the original network (like other baseline methods) to compare the performance of these methods more equitably. By modeling the network, we can obtain the probabilities that nodes’ membership in different communities, as shown in Eq (5), which can be treated as the social dimensions of nodes. Then one-vs-rest linear SVM is used for discriminative learning.

#### 2. EdgeCluster

EdgeCluster [15] is one of the most representative methods to handle multi-label classification task on large-scale network. By proposing an edge-centric k-means clustering method, it can obtain sparse social dimensions effectively, and then one-vs-rest linear SVM is used for discriminative learning. It has been shown to perform comparably as modularity maximization approaches to extract social dimensions [19] with the added advantage of scaling to large-scale network.

#### 3. Majority

By calculating the labels of all known nodes in the network, Majority predicts unknown nodes according to the proportion of different classes in labeled data. This method does not make use of the network information, but only consider of the label information.

#### 4. Random

This method predicts the node’s community membership randomly, and uses it as latent social dimensions for discriminative learning. As it can be seen, neither network structure nor label information is used in this method.

### Datasets

We tend to extract latent social dimensions by BBSD to handle multi-label classification. Three public real-world social networks are used to compare the performance of different methods.

1. *Blogcatalog* [27] Blogcatalog is a popular blog search engine and blogging community. The site’s purpose is to help bloggers connect, share ideas, and grow through group and general discussions. For a given network, bloggers categories can be measured by their articles categories. This public dataset contains 10,312 nodes, 333,983 edges and a total of 39 categories.
2. *Flickr* [27] Flickr is a social network that offers an online photo sharing network for its users. It provides its users with the ability to capture, share, and view a number of images related to many categories. This public dataset contains 80,513 nodes, 5,899,882 edges and a total of 195 categories.
3. *YouTube* [27] YouTube is a video-sharing website that allows users to upload, view and share videos. We select a subset of data from the original YouTube dataset using snowball sampling. This dataset contains 200,047 nodes, 1,275,518 edges and a total of 47 categories.

Based on the assumption of the generative process, BBSD can extract social dimensions from directed weighted graph; when the network is undirected, BBSD can also be directly applied by treating undirected edges as two directed edges.

### Evaluation Measures

In multi-class and multi-label classification task, Macro-F1 [9] and Micro-F1 [9] are commonly used to measure the classification performance. Given a network with *M* nodes, there are total *L* labels. Let *y*_*i*_ ∈ {0,1}^*L*^ is the true label sets of node *x*_*i*_, and ${\hat{y}}_{i}\in {\left\{0,1\right\}}^{L}$ is the predicted label sets of node *x*_*i*_.

- **Macro-F1.** For label *l*, Precision, Recall and F1 can be calculated as follows:
  $$\begin{array}{c} P_{l}=\frac{\sum_{i=1}^{M}y_{i}^{l}\cdot {\hat{y}}_{i}^{l}}{\sum_{i=1}^{M}{\hat{y}}_{i}^{l}},R_{l}=\frac{\sum_{i=1}^{M}y_{i}^{l}\cdot {\hat{y}}_{i}^{l}}{\sum_{i=1}^{M}y_{i}^{l}},\text{F}{\text{1}}_{l}=\frac{2P_{l}R_{l}}{P_{l}+R_{l}} \end{array}$$ (6)
  Macro-F1 firstly calculates F1 for each label, and then simply uses the average F1 value of each label to measure the performance.
  $$\begin{array}{c} \text{Macro-F1}=\frac{1}{L}\sum_{l=1}^{L}\text{F}{\text{1}}_{l}=\frac{1}{L}\sum_{l=1}^{L}\frac{2\sum_{i=1}^{M}y_{i}^{l}\cdot {\hat{y}}_{i}^{l}}{\sum_{i=1}^{M}y_{i}^{l}\text{+}\sum_{i=1}^{M}{\hat{y}}_{i}^{l}} \end{array}$$ (7)
- **Micro-F1.** Micro-F1 gives each node-label pair an equal contribution, and calculates the F-measure across all labels.
  $$\begin{array}{c} \text{Micro-F1}=\frac{2\sum_{l=1}^{L}\sum_{i=1}^{M}y_{i}^{l}\cdot {\hat{y}}_{i}^{l}}{\sum_{l=1}^{L}\sum_{i=1}^{M}y_{i}^{l}\text{+}\sum_{l=1}^{L}\sum_{i=1}^{M}{\hat{y}}_{i}^{l}} \end{array}$$ (8)

## Experiment Result

In this section, we will evaluate the performance of BBSD and four baseline methods for multi-label classification task in heterogeneous networks. We also investigate sensitivity of BBSD to the latent dimensions and hyper-parameters.

### Multi-Label Classification

In the experiment, BBSD, LDA-CD and EdgeCluster need to specify the number of social dimensions. It should be noted that, in SocioDim framework, the number of social dimensions is equal to the number of communities [10], so we use *K* to indicate the number of social dimensions as well. Through cross-validation, EdgeCluster can provide the best results when the dimension was set to 5000, 10000, and 1000 for *Blogcatalog*, *Flickr*, and *YouTube*, respectively [15]. However, if the dimension is set too large, it cannot reduce the number of features significantly. For example, setting the dimensions to 5000 will get a 10312 × 5000 social dimension matrix in *Blogcatalog* with 10312 nodes. In this paper, we aim to compare the ability of different methods to extract refined social dimension, i.e. with smaller social dimensions to obtain better performance. Therefore, we set *K* = 500 for EdgeCluster as recommended in [19] on all the three dataset, and for BBSD and LDA-CD, we set *K* = 300 on *Blogcatalog* and *Flickr*, *K* = 50 on *YouTube*. Hyper-parameters *α* and *β* in BBSD and LDA-CD were set at 50/*K* and 0.01 [28].

To evaluate the performance of different methods, dataset will be divided into training set and testing set randomly. The labels of nodes in the training set are known, and the labels of nodes in the testing set are unknown. All the classification methods need to utilize the training set to predict the labels of all nodes in the testing set. For handling multi-label classification task, we apply the typically used strategy, which assumes the number of labels of unlabeled nodes is already known and check the match of the top-ranking labels with the truth [9, 10, 15].

We choose different labeled proportion (LP) for the three dataset respectively, LP from 10% to 90% for *Blogcatalog*, and LP from 1% to 10% for *Flickr* and *YouTube*. Macro-F1 and Micro-F1 are used to measure the performance of different methods. Each classification process is repeated 10 times, and the average performance is reported in Tables 1–3.

**Table 1:** Classification performance on *Blogcatalog*.

LP	10%	20%	30%	40%	50%	60%	70%	80%	90%
Macro_F1(%)									
BBSD	**14.43±0.42**	**16.08±0.46**	**17.01±0.38**	17.51±0.39	17.65±0.48	18.07±0.67	18.04±0.48	18.05±0.87	18.30±1.02
EdgeCluster	14.25±0.36	15.77±0.27	16.85±0.31	**17.82±0.39**	**18.48±0.37**	**18.97±0.44**	**19.18±0.52**	**19.10±1.01**	**19.67±1.21**
LDA-CD	11.75±0.33	12.93±0.30	14.20±0.15	15.00±0.41	15.46±0.42	15.82±0.43	16.13±0.72	15.63±0.45	16.47±0.78
Majority	2.46±0.17	2.55±0.12	2.53±0.13	2.58±0.12	2.60±0.10	2.54±0.09	2.60±0.09	2.71±0.16	2.61±0.16
Random	4.16±0.15	4.07±0.19	4.08±0.21	4.11±0.21	4.06±0.18	4.00±0.22	4.06±0.31	4.03±0.33	4.09±0.80
Micro_F1(%)									
BBSD	24.95±0.50	**27.23±0.38**	**29.37±0.29**	**31.03±0.46**	**31.92±0.29**	**32.77±0.80**	**33.02±0.56**	**33.41±0.60**	33.40±1.28
EdgeCluster	**25.12±0.55**	26.43±0.39	28.37±0.33	30.04±0.43	31.34±0.40	32.49±0.24	32.89±0.54	33.31±0.84	**34.28±1.01**
LDA-CD	19.53±0.34	21.97±0.30	24.21±0.34	26.06±0.35	27.31±0.49	27.95±0.61	28.68±0.44	28.71±0.59	29.55±1.22
Majority	16.30±0.72	16.62±0.64	16.60±0.55	16.74±0.55	16.73±0.43	16.90±0.36	16.98±0.61	17.01±0.81	17.05±0.76
Random	4.85±0.16	4.80±0.18	4.79±0.23	4.85±0.21	4.81±0.20	4.78±0.27	4.83±0.36	4.68±0.40	4.78±0.72

**Table 2:** Classification performance on *Flickr*.

LP	1%	2%	3%	4%	5%	6%	7%	8%	9%	10%
Macro_F1(%)										
BBSD	**11.20±0.55**	**13.19±0.44**	**14.35±0.34**	**15.11±0.27**	**15.87±0.28**	**16.30±0.25**	**16.78±0.29**	**17.10±0.21**	**17.39±0.18**	**17.70±0.18**
EdgeCluster	6.91±0.21	8.21±0.24	9.02±0.22	9.57±0.30	9.95±0.27	10.22±0.17	10.51±0.10	10.63±0.18	10.77±0.21	10.87±0.15
LDA-CD	9.06±0.42	10.75±0.34	12.13±0.21	13.18±0.25	13.89±0.22	14.27±0.28	15.09±0.15	15.43±0.14	15.77±0.20	16.05±0.24
Majority	0.46±0.02	0.46±0.01	0.46±0.02	0.45±0.01	0.46±0.01	0.45±0.01	0.46±0.01	0.46±0.01	0.46±0.01	0.46±0.01
Random	0.72±0.02	0.70±0.03	0.71±0.03	0.72±0.03	0.72±0.03	0.70±0.03	0.70±0.03	0.71±0.03	0.70±0.04	0.70±0.03
Micro_F1(%)										
BBSD	**22.16±0.50**	**24.52±0.47**	**26.15±0.37**	**27.15±0.23**	**28.48±0.24**	**29.39±0.19**	**30.16±0.15**	**30.90±0.11**	**31.51±0.19**	**32.07±0.10**
EdgeCluster	19.54±0.46	20.71±0.42	22.04±0.43	22.93±0.28	23.80±0.31	24.49±0.23	25.31±0.14	25.85±0.23	26.49±0.17	27.11±0.20
LDA-CD	17.81±0.52	19.81±0.55	21.69±0.19	23.34±0.40	25.01±0.25	26.00±0.29	27.45±0.16	28.40±0.25	29.29±0.24	30.07±0.23
Majority	16.47±0.16	16.54±0.14	16.54±0.12	16.44±0.12	16.56±0.09	16.48±0.14	16.51±0.14	16.49±0.12	16.53±0.12	16.53±0.10
Random	0.94±0.02	0.93±0.04	0.93±0.03	0.94±0.03	0.94±0.03	0.93±0.03	0.92±0.04	0.93±0.04	0.92±0.03	0.92±0.03

**Table 3:** Classification performance on *YouTube*.

LP	1%	2%	3%	4%	5%	6%	7%	8%	9%	10%
Macro_F1(%)										
BBSD	**21.37±1.28**	**26.09±1.08**	**28.15±0.81**	**29.28±0.45**	**30.04±0.55**	**31.27±0.37**	30.94±0.61	31.54±0.51	31.81±0.58	31.90±0.52
EdgeCluster	19.67±1.43	22.94±0.92	23.68±0.78	25.81±0.80	28.11±1.16	30.07±0.78	**31.00±1.02**	**32.20±0.58**	**32.99±0.77**	**33.49±0.79**
LDA-CD	21.28±0.77	24.55±1.03	26.41±0.64	27.52±0.30	27.79±0.24	28.95±0.16	28.85±0.36	29.51±0.56	29.43±0.31	29.50±0.46
Majority	7.80±0.46	7.61±0.59	7.69±0.47	7.74±0.51	7.61±0.34	7.72±0.40	7.74±0.20	7.62±0.26	7.59±0.27	7.73±0.28
Random	7.27±0.08	7.13±0.10	7.13±0.13	7.14±0.15	7.19±0.15	7.16±0.15	7.14±0.17	7.14±0.11	7.17±0.11	7.09±0.15
Micro_F1(%)										
BBSD	**28.44±1.00**	**34.50±1.20**	**37.19±0.98**	**38.46±0.52**	**39.43±0.70**	**40.80±0.39**	**40.85±0.77**	41.62±0.59	41.65±0.66	42.07±0.42
EdgeCluster	22.92±2.56	30.64±0.65	32.63±0.58	35.42±0.94	37.92±0.74	39.31±0.57	40.55±0.75	**41.81±0.38**	**42.55±0.48**	**43.10±0.54**
LDA-CD	27.13±1.46	32.16±0.99	34.69±0.64	35.93±0.36	36.98±0.49	38.01±0.46	38.35±0.40	38.56±0.46	38.87±0.45	39.24±0.44
Majority	26.82±1.41	27.17±0.90	27.42±0.62	27.56±0.67	27.45±0.34	27.59±0.50	27.64±0.26	27.58±0.19	27.63±0.31	27.73±0.22
Random	7.64±0.11	7.50±0.10	7.54±0.13	7.52±0.17	7.58±0.14	7.53±0.16	7.52±0.15	7.53±0.09	7.54±0.09	7.46±0.14

We can find that on *Blogcatalog* dataset, BBSD achieves competing performance with EdgeCluster, and 3%—5% higher performance than LDA-CD. On *Flickr* dataset, BBSD gets 5% higher performance than EdgeCluster, and outperforms LDA-CD by 2% at higher labeled proportion and 5% at lower labeled proportion. On *YouTube* dataset, BBSD achieves 3% higher results than LDA-CD, and outperforms EdgeCluster at lower labeled proportion on both macro-F1 (2%) and micro-F1 (5%). Majority and Random perform extremely poorly on all the three datasets.

As it can be seen, by using smaller social dimensions, BBSD can obtain satisfactory classification results. The reasons mainly lie on the following aspects.

Firstly, experimental datasets are crawled from real-world networks. In the crawling process, information will be lost inevitably. For example, in real-world networks, an edge exists between node A and node B. But in the crawling process, the edge may be lost. As no relationship exists between node A and node B in the crawled network, it may lead to wrong community assignment for these nodes and yield poor performance. BBSD models the network from the perspective of the probability to connect communities, which considers all the possible behaviors of nodes. When the edges are missing, BBSD can reduce the impact of noise data with the advantage of prior probability, and obtain more satisfactory results.

Secondly, in order to improve efficiency, EdgeCluster requires that the social dimensions of each node should not exceed its degree, and then it will obtain relatively sparse social dimension matrix. However, when community detection algorithms fail, sparse matrix may be unable to represent all possible social dimensions accurately, leading to lower classification results. Due to data loss and other reasons in the crawling process, the current observed network may be different from the real-world network. Similarly, from the evolutionary perspective, the current network is just a status of the real-world network. Therefore, the observed behaviors in the current network may not reflect the real behaviors of nodes. Our method does not restrain the social dimensions of each node, which ensures to mine all the possible interaction behaviors between nodes and communities, making it closer to the behavior feature in real-world network and yield better results. Meanwhile, BBSD tries to improve efficiency by setting smaller number of social dimensions, which can reduce the size of social dimension matrix and improve the efficiency for discriminative learning greatly.

Finally, community detection methods will fail when setting improper parameters. For example, when setting smaller social dimensions, nodes with different labels may be included in the same communities. In this case, community detection based methods cannot extract social dimensions accurately, making the classification performance drop. Behavior-based methods do not consider the nodes’ membership in communities. So even the node to be predicted is assigned to wrong communities, it won’t affect the classification results. In addition, our method relies on similarity of behavior features, rather than the quality of community detection. As long as the behavior features are similar, nodes will be predicted to be the same category. Therefore, BBSD has a strong fault-tolerant ability for community detection algorithm, which can obtain more satisfactory classification performance when some community detection algorithms fail.

EdgeCluster often require larger social dimensions for classification. When choosing smaller dimensions, EdgeCluster is unable to extract the social dimensions accurately, making the classification performance drop. Due to differences in text and network, LDA-CD cannot detect community accurately [26], so in this case, the extracted social dimensions are less discriminative, leading to poor classification performance as well. Majority and Random fail because they do not make full use of network information for learning.

Another observation is that BBSD gets more satisfactory performance on *Flickr*. Through comparative analysis we can find that the number of edges in *Flickr* dataset is relatively larger, i.e. we can observe more behaviors of nodes. Therefore, BBSD can distinguish behavior feature of the different nodes better in the modeling process, resulting in more satisfactory results.

### Parameter sensitivity

#### Sensitivity to social dimensions

The performance of community detection algorithm varies with different social dimensions, which affects the subsequent discriminative classifiers directly. In the era of big data, the network becomes much larger. When computing resources are limited, it is necessary to reduce the nodes’ feature to smaller dimensions. In this section, we evaluate the classification performance of different methods when setting different social dimensions.

We vary the number of social dimensions (from 50 to 500) in the experiment and compare the performance of BBSD, EdgeCluster and LDA-CD on all the three data sets. In order to avoid the influence of labeled proportion, we set two different labeled proportions on each dataset (50% and 90% for *Blogcatalog*, 5% and 9% for *Flickr*, 5% and 9% for *YouTube*). The experiment results are shown in Tables 4–9.

**Table 4:** Parameter sensitivity to social dimensions on *Blogcatalog* (LP = 50%).

Dimensions K	50	100	200	300	400	500
Macro_F1(%)						
BBSD	**12.05±0.40**	**14.72±0.41**	**16.48±0.47**	**18.15±0.60**	**18.22±0.41**	17.90±0.45
EdgeCluster	9.05±0.28	11.80±0.18	14.67±0.46	15.90±0.47	17.42±0.39	**18.55±0.48**
LDA-CD	10.05±0.33	12.86±0.26	14.79±0.44	15.68±0.36	15.00±0.42	15.67±0.46
Micro_F1(%)						
BBSD	**29.75±0.33**	**31.63±0.34**	**31.68±0.47**	**32.45±0.36**	**31.26±0.30**	30.34±0.49
EdgeCluster	24.75±0.28	27.56±0.22	29.90±0.65	30.66±0.54	31.03±0.35	**31.29±0.49**
LDA-CD	24.71±0.24	27.31±0.48	27.69±0.42	27.37±0.47	25.60±0.26	25.11±0.46

**Table 5:** Parameter sensitivity to social dimensions on *Blogcatalog* (LP = 90%).

Dimensions K	50	100	200	300	400	500
Macro_F1(%)						
BBSD	**11.95±0.47**	**14.58±1.26**	**16.68±0.88**	**17.91±1.19**	18.01±1.29	18.35±1.13
EdgeCluster	9.12±0.49	11.73±0.47	14.42±0.72	16.14±0.70	**18.55±1.05**	**19.75±0.98**
LDA-CD	10.04±0.72	12.71±0.95	15.15±1.00	15.84±0.96	15.94±0.59	16.73±0.93
Micro_F1(%)						
BBSD	**29.66±1.02**	**31.99±1.20**	**33.01±1.26**	**33.66±1.26**	32.95±1.23	32.99±1.66
EdgeCluster	25.34±0.87	28.01±0.61	30.49±0.95	32.72±0.84	**33.55±0.77**	**34.39±1.12**
LDA-CD	25.24±0.89	27.96±0.79	29.58±1.49	29.00±1.32	28.60±0.74	28.44±1.41

**Table 6:** Parameter sensitivity to social dimensions on *Flickr* (LP = 5%).

Dimensions K	50	100	200	300	400	500
Macro_F1(%)						
BBSD	**10.75±0.22**	**13.55±0.17**	**14.85±0.30**	**15.81±0.28**	**16.62±0.32**	**16.41±0.28**
EdgeCluster	5.37±0.18	6.37±0.18	7.54±0.12	8.53±0.21	9.25±0.18	10.02±0.05
LDA-CD	8.83±0.20	11.66±0.19	13.24±0.13	13.86±0.22	14.11±0.38	13.49±0.40
Micro_F1(%)						
BBSD	**30.74±0.16**	**31.69±0.18**	**29.69±0.29**	**28.41±0.19**	**27.94±0.29**	**27.37±0.20**
EdgeCluster	24.34±0.13	24.96±0.20	24.34±0.18	23.79±0.21	23.62±0.29	23.82±0.18
LDA-CD	29.16±0.07	30.17±0.11	27.48±0.37	24.89±0.28	23.20±0.23	22.26±0.26

**Table 7:** Parameter sensitivity to social dimensions on *Flickr* (LP = 9%).

Dimensions K	50	100	200	300	400	500
Macro_F1(%)						
BBSD	**10.72±0.21**	**14.09±0.25**	**16.22±0.21**	**17.43±0.19**	**18.04±0.30**	**18.06±0.26**
EdgeCluster	5.41±0.13	6.33±0.10	7.98±0.12	9.09±0.12	9.87±0.18	10.84±0.19
LDA-CD	8.87±0.11	12.02±0.19	14.68±0.13	15.88±0.16	16.29±0.19	15.45±0.24
Micro_F1(%)						
BBSD	**31.23±0.10**	**33.03±0.11**	**32.61±0.11**	**31.54±0.10**	**30.74±0.21**	**30.09±0.23**
EdgeCluster	24.84±0.10	26.11±0.10	26.97±0.14	26.76±0.22	26.70±0.26	26.53±0.16
LDA-CD	29.59±0.14	31.64±0.12	31.23±0.19	29.44±0.16	27.53±0.20	25.66±0.28

**Table 8:** Parameter sensitivity to social dimensions on *YouTube* (LP = 5%).

Dimensions K	50	100	200	300	400	500
Macro_F1(%)						
BBSD	**30.33±0.51**	**29.67±0.48**	27.30±0.50	25.61±0.53	26.20±0.54	25.30±0.45
EdgeCluster	26.01±0.84	28.49±0.51	**30.20±0.82**	**30.12±0.47**	**29.44±1.18**	**28.13±0.84**
LDA-CD	27.93±0.78	28.71±0.80	26.62±0.47	24.06±0.74	23.09±0.74	22.02±0.68
Micro_F1(%)						
BBSD	**40.06±0.67**	37.72±0.69	34.37±0.42	31.90±0.60	31.72±0.80	31.02±0.79
EdgeCluster	39.74±0.57	**40.51±0.27**	**40.88±0.47**	**39.63±0.66**	**38.58±0.73**	**37.76±0.44**
LDA-CD	36.84±0.83	36.41±0.51	32.91±0.53	29.77±0.71	27.97±0.53	26.84±0.47

**Table 9:** Parameter sensitivity to social dimensions on *YouTube* (LP = 9%).

Dimensions K	50	100	200	300	400	500
Macro_F1(%)						
BBSD	**31.60±0.48**	**32.17±0.64**	31.15±0.48	29.16±0.36	29.10±0.49	28.07±0.34
EdgeCluster	26.74±0.34	29.31±0.43	**31.63±0.32**	**33.20±0.57**	**33.04±0.48**	**33.18±0.59**
LDA-CD	29.50±0.46	30.67±0.26	29.75±0.31	28.05±0.43	27.18±0.40	25.27±0.65
Micro_F1(%)						
BBSD	**41.55±0.57**	40.67±0.42	38.52±0.46	36.06±0.38	35.59±0.57	34.81±0.48
EdgeCluster	40.85±0.24	**41.90±0.26**	**42.99±0.25**	**43.04±0.39**	**42.75±0.40**	**42.59±0.35**
LDA-CD	38.86±0.19	38.87±0.53	37.05±0.21	34.62±0.55	33.11±0.48	31.07±0.73

It can be seen that on *Flickr* dataset, the performance of BBSD is significantly higher than other methods. EdgeCluster performs much poor when setting the same dimensions, it has shown 4%—8% lower performance than BBSD; the same situation also occurred in smaller dimensions on *Blogcatalog* and *YouTube* (*K* < 400 for *Blogcatalog*, and *K* < 200 for *YouTube*), BBSD can still get the best performance. However, when selecting larger dimensions on *Blogcatalog* and *YouTube* (*K* = 500 for *Blogcatalog*, and *K* > 100 for *YouTube*), EdgeCluster’s performance began to exceed BBSD, indicating that EdgeCluster requires more social dimensions to extract the latent features of nodes. In the area of big data, when we try to get satisfactory results by relatively limited computing resources, or when the limited computing resources only allows choosing smaller dimensions, BBSD can extract refined social dimensions accurately based on nodes’ behavior feature, and obtain better classification performance.

#### Sensitivity to hyper-parameters

**Fig 3:**
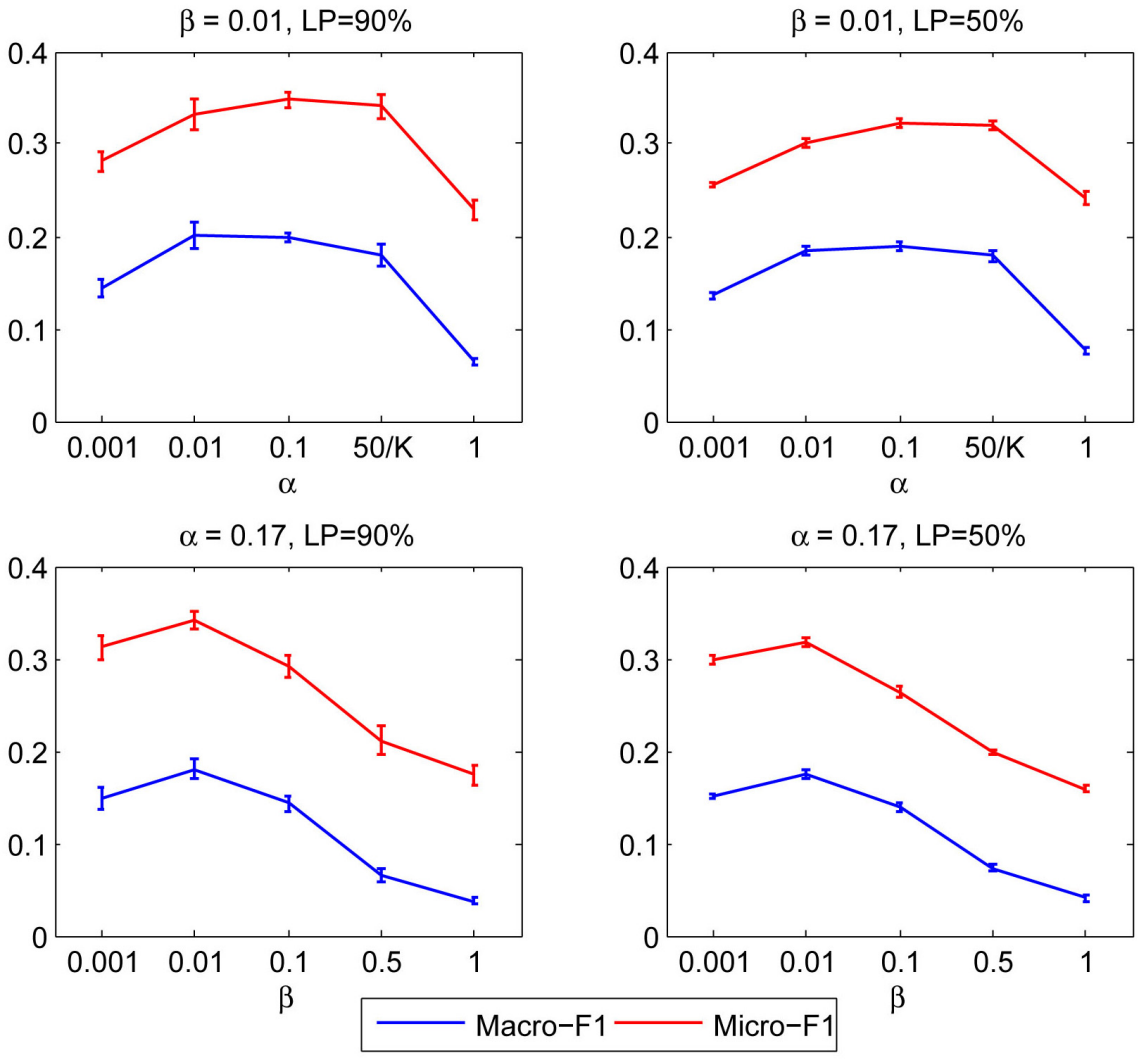
Performance of BBSD on different hyper-parameters.

Hyper-parameters are used to integrate prior knowledge and avoid over-fitting. In order to evaluate the impact of hyper-parameters, we fix dimensions *K* = 300 and select two different labeled proportions (LP = 50% and 90%) on *Blogcatalog* dataset. Firstly, we fix *β* = 0.01 and vary *α* from 0.001 to 1, the results are shown in Fig (3a) and (3b); then we fix *α* = 0.17 and vary *β* from 0.001 to 1, the results are shown in Fig (3c) and (3d).

As it can be seen, setting different hyper-parameters does affect classification performance. Hyper-parameters need to be constricted in a reasonable range, which should not be set too large or too small. This is because the larger hyper-parameters would weaken the observed behaviors, making the discrimination of behavior features insufficient; Smaller hyper-parameters are able to highlight the importance of observed data, but will make the behavior feature concentrated in certain communities, which are easily affected by noise data. In practice, the optimal hyper-parameters depend on the attribute of different data sets and can be determined by cross validation.

## Conclusion

By extracting latent social dimensions based on network connectivity information, SocioDim framework can deal with the multi-label classification task in heterogeneous networks effectively. However, traditional methods mostly use community detection algorithms to extract latent social dimensions, and often require larger dimensions to obtain satisfactory results. In the era of big data, the size of network is becoming much larger, the feature of nodes needs to be reduced to smaller dimensions, so that an ordinary computer can perform classification task in large-scale network.

In this paper, we propose a novel social dimension extraction method, BBSD, to handle multi-label classification task in large-scale network. Instead of using community detection, BBSD tries to extract the nodes’ behavior features with different communities, which are used as latent social dimensions for classification. Compared to community detection methods, BBSD relies on the community distributions of neighbor nodes, rather than node’s membership in communities. So even the node to be predicted is assigned to the wrong communities, it won’t affect the classification performance of BBSD, which has a strong fault-tolerant ability for community detection algorithm. In addition, our method models the network from the perspective of the probability to connect communities, which considers all the possible behaviors of nodes. This strategy ensures denser social dimension matrix that is more discriminative, and can effectively reduce the effect of noise data. Experiments on a variety of networks illustrate our method can extract the refined social dimension accurately and obtain satisfactory classification performance with smaller dimensions.

In order to handle large-scale network, Edgecluster method restrains the number of affiliations one user can participate in is upperbounded by the number of its connections. Our method can be improved with the similar idea. When social dimension matrix is obtained, elements lower than a threshold can be eliminated to get sparse social dimensions, which can greatly improve the efficiency in the discriminative classifier. In addition, SocioDim framework is sensitive to the number of social dimensions, so we also focus on applying nonparametric methods to determine the number of dimensions automatically in further research.
